# Radar Resolution Enhancement Based on Burg-Aided MIMO-DBS and Burg-Aided MIMO-SAR †

**DOI:** 10.3390/s26092698

**Published:** 2026-04-27

**Authors:** Muge Bekar, Ali Bekar, Anum Pirkani, Christopher John Baker, Marina Gashinova

**Affiliations:** 1 Department of Electronic, Electrical and Systems Engineering, University of Birmingham, Birmingham B15 2TT, UK; a.a.a.pirkani@bham.ac.uk (A.P.); c.j.baker.1@bham.ac.uk (C.J.B.); m.s.gashinova@bham.ac.uk (M.G.); 2 Department of Electrical and Electronics Engineering, Abdullah Gul University, Kayseri 38080, Türkiye; 3 Department of Electrical and Electronics Engineering, Nigde Omer Halisdemir University, Nigde 51240, Türkiye; abekar@ohu.edu.tr

**Keywords:** multiple-input multiple-output (MIMO) radar, Doppler beam sharpening, synthetic aperture radar (SAR), back-projection, Burg algorithm, autoregressive methods, millimeter wave radar, radar imaging

## Abstract

Autonomous systems require sensors that provide high-resolution imagery in adverse lighting and weather conditions for advanced situational awareness. In this regard, radars are a mandatory component of autonomous systems. Although Multiple-Input Multiple-Output (MIMO) radars provide high angular resolution beyond that of their actual physical dimension, much higher cross-range resolutions are required, especially in traffic congested areas, to differentiate and recognize closely positioned targets. The motion of the MIMO radar platform can be exploited to obtain higher cross-range resolution in the off-boresight direction, using Synthetic Aperture Radar (SAR) and Doppler Beam Sharpening (DBS) techniques, but improvements in the boresight direction, the most crucial direction for path planning, require the use of super-resolution techniques. This paper proposes a technique that combines the Burg algorithm with MIMO-SAR and MIMO-DBS radar data to enhance the cross-range resolution in the boresight direction and to achieve further enhanced cross-range resolution in off-boresight directions. The proposed technique is applied to both frequency domain and time domain data in back-projection (BP) and DBS image formation processing. A comprehensive comparison is made, with evaluation of corresponding performance and operational complexity. The performance of the technique is validated through simulation, lab-based and real-world experiments at a frequency of 77 GHz.

## 1. Introduction

Robustness to weather and lighting conditions as well as an ability to provide actual measurements of target parameters have made a radar a compulsory sensor to enable high levels of automation. However, for full autonomy, radar sensors should be capable of providing high-fidelity imagery of the scene within the radar field of view, which enables segmentation, classification and, therefore, path planning. The industrial requirement of the sensor to be of a compact size and low cost led to wide adoption of wide bandwidth Frequency Modulated Continuous Wave (FMCW) mm-wave waveforms and Multiple-Input Multiple-Output (MIMO) beamforming principle [1], which allows sensor dimensions to be compact enough to fit into a densely packed vehicle infrastructure. While widely available bandwidth is already suitable for radar imagery, cross-range resolution needs improvement beyond that provided by MIMO sensors. Moreover, the ability to image the whole scene within the radar field of view requires beamforming with no or little degradation in the lateral (off-boresight) directions. MIMO arrays form a virtual aperture with significantly fewer physical antenna elements, compared to a uniform phased array for the same resolution. However, similar to electronical scanning of phased arrays, the length of the effective MIMO aperture decreases at lateral directions, widening the MIMO beam and, accordingly, coarsening the resolution.

To enhance cross-range resolution using radar platform motion, the Synthetic Aperture Radar (SAR) and Doppler Beam Sharpening (DBS) techniques have been adopted for automotive radars. The DBS, originally proposed for airborne radar and often referred as an “unfocussed SAR” [2,3,4], has been shown to deliver high-resolution automotive imagery at sub-THz frequencies [5,6].

The technique uses different Doppler shifts of the same scatterer when it moves through the radar beam during the platform motion. The wider the beam, the larger the Doppler spread and, therefore, based on the Doppler resolution, the position of the scatterer can be mapped in azimuth at refined angular directions, with the refinement factor proportional to the frequency and the speed of the platform [5]. Specificity of automotive domain is a radar mostly looking in the forward direction. The DBS, when applied to such data, will suffer from an ambiguity related to the side-agnostic Doppler shifts on either the right or the left sides from the boresight. This ambiguity, however, can be readily removed by a combination of DBS with MIMO beamforming (MIMO-DBS), as shown in [7,8,9,10], so that the true angular position of targets is determined. Importantly, in addition to resolution of side ambiguity, a notable enhancement in cross-range resolution at lateral directions compared to that of pure MIMO is achieved with the further benefits of a significant reduction in the sidelobe levels inherent to the pure MIMO beamforming.

Side-looking SAR has also been used to obtain high-resolution images of road infrastructure, such as parking slots and curbs [11,12,13,14,15]. Subsequently, a forward-looking MIMO-SAR was proposed and its performance was studied in [16,17,18,19]. In [16], an autofocus algorithm was used to estimate and compensate for motion errors that occur during the MIMO-SAR integration time, and in [17], deep learning and back-projection (BP) techniques to form a 2-D radar image with enhanced angular resolution have been proposed. However, even in advanced MIMO-SAR, the cross-range resolution in the boresight (straight ahead) direction is still defined by the achievable boresight MIMO resolution, limiting the ability of radar to differentiate closely positioned objects at the front.

In this paper, we propose the use of the Burg algorithm (BA) to both interpolate and extrapolate the data gathered during the motion of a MIMO radar in two dimensions: the Doppler dimension, along the synthetic aperture, and spatial dimension, along the MIMO virtual array aperture, so that angular resolution can be enhanced beyond that of MIMO radar in both the forward-looking and the lateral directions. The BA, widely used and well presented in the literature, is selected for this study due to its computational efficiency, speed, ease of implementation, and ability to enhance resolution by up to a factor of three [20,21,22,23]. In [24], the BA was employed to predict missing data in SAR imagery, resulting in a notable improvement in the image quality. In [25,26], the BA was combined with DBS to enhance cross-range resolution, while in [27,28,29], it was combined with 1-D and 2-D MIMO to both reconstruct missing data by the interpolation and enhance the resolution by extrapolation of the virtual array elements.

In this paper we present a comprehensive study of combined MIMO-SAR and MIMO-DBS with BA, with a main focus on the quantitative evaluation and comparison of the performance versus the operational and computational complexity of each technique. We believe this work, stemming from the authors’ previous works [19,26,28,29,30], will be useful in advancing automotive radar imagery without hardware modification or use of greedy resource-consuming algorithms.

The proposed methods require no prior knowledge of the target area whilst ensuring resolution enhancement. The main contributions of this article are as follows:

(1) For the first time, the Burg algorithm is applied to the data in three domains—range, Doppler, and angle—and the processing steps are presented within a common framework.
(2) The proposed method is investigated through the image formation techniques of MIMO-DBS and back-projection MIMO-SAR, which are processed in the frequency and time domains, respectively. The performance and computational complexity of each approach is comparatively analyzed to demonstrate its respective advantages and disadvantages.
(3) The validation and analysis are conducted through simulations, lab-based and real-world experiments, using an off-the-shelf 1-D MIMO radar operating at 77 GHz.

The rest of the paper is organized as follows: Section 2 gives a detailed description of MIMO radar, DBS, MIMO-DBS, and BP for forward-looking MIMO-SAR radar, as well as the Burg algorithm. Section 3 then presents the proposed combined methods and evaluates their computational complexity. The simulation and experimental results are discussed in Section 4 and Section 5, respectively. Conclusions are outlined in Section 6.

## 2. Background

For the integrity and benchmarking analysis within a common notation and theoretical framework, a brief description of MIMO, DBS, MIMO-DBS, BP for MIMO-SAR, and the Burg algorithm is presented in this section. Without loss of generality on the whole concept, here we use a FMCW waveform due to its widespread use in radar systems and our experiments.

The up-chirp transmitted FMCW signal is expressed as [31]

(1) $$s\left(t\right)=e^{j(\phi +2\pi f_{c}t+\pi \gamma t^{2})},\;0\leq t<T_{m}$$

where ϕ is the initial phase, $f_{c}$ is the carrier frequency, and γ is the chirp rate, defined as $\frac{B}{T_{m}}$, where B and $T_{m}$ are the bandwidth and the sweep time, respectively.

The received echo from a target at the range of R is

(2) $$s_{R}\left(t\right)=e^{j(\phi +2\pi f_{c}(t-\tau )+\pi \gamma {\left(t-\tau \right)}^{2}+\alpha (t))}$$

where $\tau =\frac{2R}{c}$ is the delay time, c is the speed of light and α(t) is an additional phase due to a Doppler frequency shift, $f_{d}$.

The Fourier transform of the intermediate frequency (IF) signal, $s_{IF}\left(t\right)$, yields the range compressed signal:

(3) $$D\left(f_{r}\right)=\int_{0}^{T_{m}}s_{IF}\left(t\right)e^{-j2\pi f_{r}t}dt≅sinc\left[T_{m}\left(f_{r}-\gamma \tau \right)\right]$$

where $f_{r}$ are the range-frequency samples.

### 2.1. MIMO Radar

**Figure 1:**
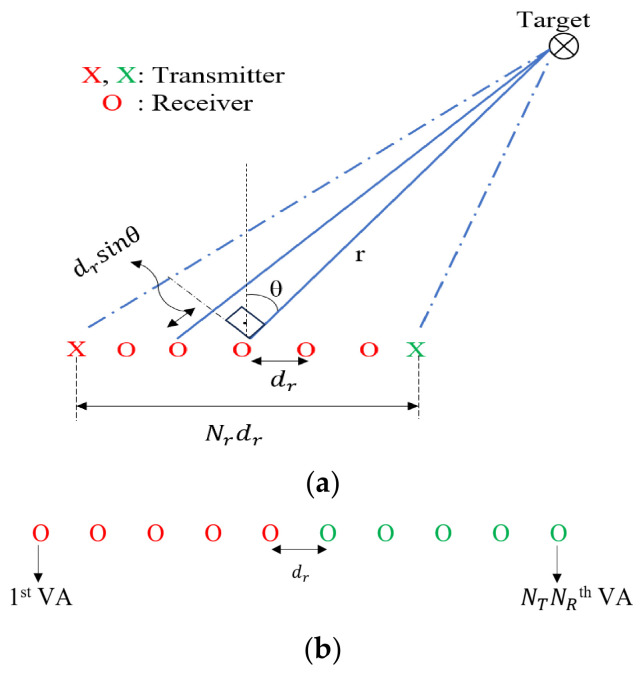
Sketch of (**a**) a conventional MIMO array configuration, (**b**) the virtual array configuration of (**a**).

We will assume here that the conventional MIMO array consists of $N_{T}$ physical transmit elements (Tx) and $N_{R}$ physical receive elements (Rx), and each pair of Tx and Rx form a virtual element of a virtual MIMO array of the size $M=N_{T}\cdot N_{R}$, as shown in Figure 1, where $d_{r}$ is the spacing between elements. The received signal from a target, positioned at the far-field region of the array at an azimuth angle θ from the boresight, after the range compression at each virtual element, is defined as

(4) $$D\left(r,m\right)=D\left(r\right)e^{j\left(m-1\right)(2\pi /\lambda )d_{r}sin\theta }$$

where λ is wavelength, m is the number of the virtual element, m=1, 2,⋯, M, and $r=\frac{{cf}_{r}}{2\gamma }$ is related to the sampled range bin. The angular resolution is determined by the length of the virtual array, $L=d_{r}(N_{T}N_{R}-1)$, and the cosine of the target’s angular position [31]:

(5) $$\theta_{-3dB}=0.88\frac{\lambda }{Lcos\theta }$$

showing widening of the beam away from the boresight.

### 2.2. Doppler Beam Sharpening

**Figure 2:**
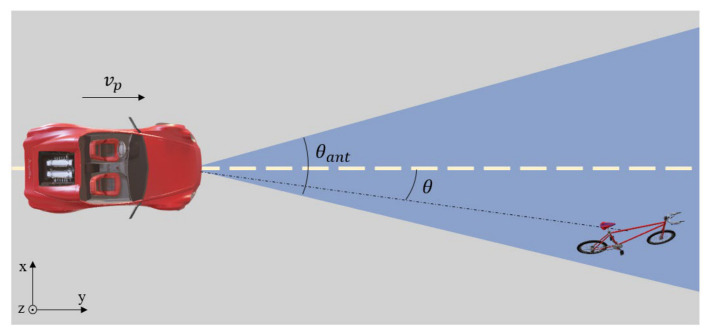
Geometry of forward-looking DBS.

When a radar system is in motion, the Doppler frequency of the scattering point on the target, shown in Figure 2, changes with the change of angle θ between the platform velocity vector and line of sight to the scatterer [5]:

(6) $$f_{d}=\frac{2v_{r}}{\lambda }=-\frac{2{|v}_{p}|cos\theta }{\lambda }$$

where $v_{p}$ is the platform velocity and $v_{r}$ is radial velocity.

The relationship between the angular resolution and the target’s angle in the DBS technique is given by [7]

(7) $$\theta_{DBS\_FL}≅\frac{0.9\lambda }{2Tv_{p}sin\theta }$$

where $T=NT_{PRI}\;$ is the total coherent processing interval (CPI), showing improvement in the resolution with an increase of θ, in contrast to the MIMO trend expressed by (5).

The 2-D received signal after range compression at each Doppler sample point is

(8) $$D(r,n)≅sinc\left[T_{m}\left(f_{r}-\gamma \frac{2R_{n}}{c}\right)\right]e^{j\left(n-1\right)\left(\frac{2\pi }{\lambda }\right)v_{p}T_{PRI}cos\theta }$$

where $T_{PRI}$ is the pulse repetition interval, $R_{n}=R_{0}-\left(n-1\right)v_{p}T_{PRI}cos\theta$ for a forward-looking geometry. $R_{0}$ is the initial distance between the radar and the target and n=1, 2,⋯, N is the frame number, where the frame is defined as a compressed signal after $n^{th}$ integration.

The use of DBS does not allow for distinguishing between right and left side within the field of view (FoV), so that the angular direction with respect to the boresight becomes ambiguous. Also, when the radar platform velocity is higher than ${2v}_{max}$, where $v_{max}=\frac{\lambda }{4T_{PRI}}$ ([7,32]), the ambiguities in angle occur. The unambiguously resolvable maximum angle, $\theta_{max}$, to the stationary target is

(9) $$\theta_{max}=\left\{\begin{array}{cc} \arccos\left(1-\frac{2v_{max}}{v_{p}}\right), & if\;v_{p}>{2v}_{max}\\ 90{}^{\circ}, & otherwise \end{array}\right.$$

**Figure 3:**
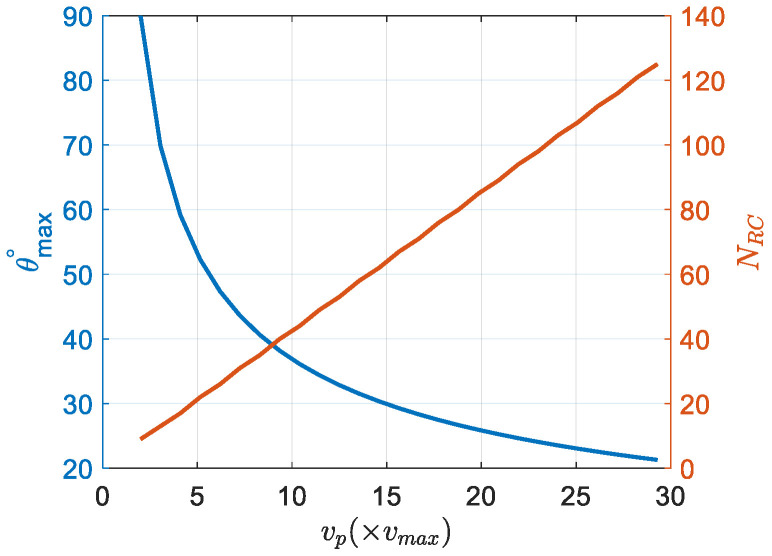
The achievable maximum unambiguous angle and the number of range cells versus platform velocity.

Figure 3 (left axis) shows the decrease in the unambiguous field of view $\theta_{max}$ as the platform velocity increases. In order to increase the maximum unambiguous angle, in [33], the authors proposed an approach to use reconstructed data with the multiple replicas of the actual radar data.

During DBS integration, the scatterers in the scene may appear in different range bins due to motion-related range cell migration (RCM), which can be simply estimated as a ratio of distance travelled by the radar to the range resolution (ΔR):

(10) $$N_{RC}=\frac{Tv_{p}cos\theta }{\Delta R}=\frac{Tv_{p}cos\theta }{\left(c/2B\right)}$$

where $N_{RC}$ is the number of range cells and B is the signal bandwidth.

Figure 3 (right axis) shows the relationship between the number of range cells and the platform velocity for a scatterer at the boresight, calculated for T = 128 ms and ΔR = 30 cm.

To mitigate the RCM, when the collected energy is spread across multiple range bins, causing a smearing and defocusing of the radar image, either range cell migration correction (RCMC), as in [34], or a reduction in the effective bandwidth by use of weighting coefficients, can be used. The latter case would inevitably lead to a coarsening of the resolution, though it would still be a suitable measure for cases of relatively small RCM and when the range resolution is much finer than the angular resolution, so that a slight sacrifice in resolution would be tolerable.

The DBS is based on the Fast Fourier transform (FFT) to process received signals after range compression, making DBS processing much faster than back-projection in SAR, as will be shown in Section 3. To apply the FFT, the phase of the signal must change linearly; however, as the aperture length becomes large, the phase variation can become non-linear. According to [35], the maximum allowable phase variation for effective FFT is π/2, after which it results in defocused images, so that further processing may be needed to correct the phase non-linearity.

### 2.3. MIMO-DBS Technique

The combination of MIMO and DBS for forward-looking directions overcomes the shortcomings of each individual MIMO and DBS by the enhancement of angular resolution in the lateral directions, compared to MIMO, significant reduction in MIMO sidelobe levels, and removal of the right and left side spatial ambiguity inherent to DBS and SAR [7,9].

The 3-D range compressed signal can be written by combining (4) and (8) as

(11) $$D\left(r,n,m\right)≅sinc\left[T_{m}\left(f_{r}-\gamma \frac{2R_{n,m}}{c}\right)\right]e^{j\frac{2\pi }{\lambda }\left(\left(n-1\right)v_{p}T_{MIMO}cos\theta +\left(m-1\right)d_{r}sin\theta \right)}$$

where $T_{MIMO}$ is the MIMO frame interval.

After range compression, FFTs along the Doppler sampling points and along the virtual array sampling points result in the formation of image domain data cube, $F\left(r,p,q\right)$:

(12) $$F\left(r,p,q\right)=\sum_{n=1}^{N}\sum_{m=1}^{M}D\left(r,n,m\right)e^{\frac{-j2\pi np}{N}}e^{\frac{-j2\pi mq}{M}}$$

where p=1:N  represents Doppler sample indices whereas q=1:M represents MIMO angular samples.

Then, in order to form the sharpened image, the 3-D radar data cube is reduced to 2-D data by selecting the intersecting samples along both MIMO and DBS dimensions [7], and the combined MIMO-DBS has an approximate −3 dB beamwidth of

(13) $$\theta_{-3dB_{MIMO-DBS}}\approx \frac{0.9}{\sqrt{{\left(\frac{\left(M-1\right)d_{r}}{\lambda }cos\theta \right)}^{2}+{\left(\frac{2Tv_{p}sin\theta }{\lambda }\right)}^{2}}}$$

It is noteworthy that for a stationary case, the resolution in (13) converges to that of MIMO, whereas when M=1, the equation gives the angular resolution of the DBS when the platform moves.

An improvement in angular resolution is achieved for targets located at angles greater than $\theta^{\prime }$:

(14) $$\theta^{\prime }=\arctan\left(\frac{(M-1)d_{r}}{2Tv_{p}}\right)$$

and further improves with the increase in the off-the-boresight angle. The resolution would also improve with the increase in the total coherent processing interval, T, but in parallel it may lead to the RCM and therefore T should be traded off in an adaptive manner per each scenario and radar parameters.

In the case of time-division multiplexing (TDM) MIMO, platform motion induces phase errors which should be compensated prior to beamforming. Assuming a constant velocity, the phase error for the u^th^ transmitter is

(15) $$∆\beta =\frac{4\pi v_{r}T_{PRI}u}{\lambda }$$

### 2.4. Back-Projection

The equation of BP to process MIMO-SAR data can be written as

(16) $$S\left(k,l\right)=\sum_{m=1}^{M}\sum_{n=1}^{N}D\left(\left[R_{klmn}\right],m,n\right)e^{-j\frac{4\pi }{\lambda }R_{klmn}}$$

where

(17) $$R_{klmn}=\sqrt{{\left(v_{p}T_{MIMO}n-x_{k}\right)}^{2}+{\left(d_{r}\left(m-1\right)-\left(M-1\right)d_{r}/2-y_{l}\right)}^{2}}$$

D is the range-compressed data, M is the total number of virtual array elements, N is the number of sampling points along the Doppler dimension, k and l are pixel numbers of the 2D image along the x and y dimensions, respectively, $R_{klmn}$ is the range between the pixel at ($x_{k},\;y_{l}$) and the radar’s $m^{th}$ virtual element at the $n^{th}$ radar position, and $\left[R_{klmn}\right]$ is the range cell number.

**Figure 4:**
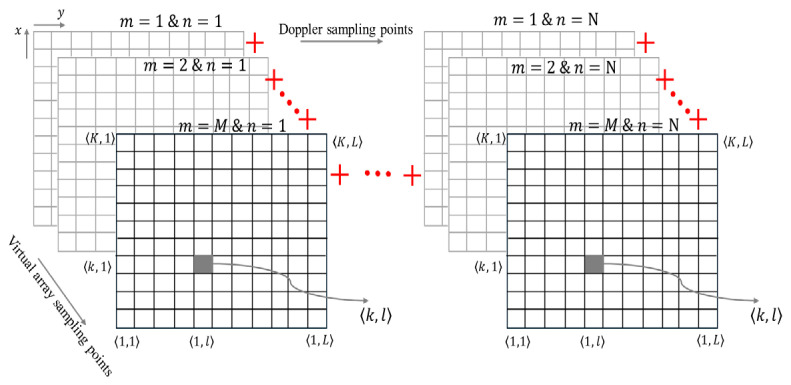
Demonstration of back-projection of MIMO-SAR.

The data formation of MIMO-SAR is illustrated in Figure 4. It should be noted that the achievable angular resolution is the same as the theoretical resolution given by (13). This will be verified through simulations and experiments in the following sections.

Unlike the DBS and MIMO-DBS techniques, back-projection does automatically account for RCM during image formation; hence, migrations do not lead to defocusing. Furthermore, BP does not encounter the limitations associated with the FFT in DBS-based techniques, such as the phase variation of radar data described in Section 2.2. Another limitation related to the uniform sampling requirement to perform FFT in the DBS approach is also overcome if the accurate Inertial Measurement Unit (IMU) data is available: the BP algorithm can handle non-linear motion by directly incorporating the precise position and orientation information.

For clarity, the advantages and disadvantages of both techniques are summarized in Table 1. It is worth stressing that while MIMO-SAR would be superior for high-accuracy image formation, the main advantage of MIMO-DBS remains its computational efficiency, allowing near real-time image formation, due to very significant gains in the computational time and required resources, as will be detailed in Section 3.3.

### 2.5. Burg Algorithm

In phase arrays or MIMO, the aperture can be synthetically increased by extrapolation, resulting in higher angular resolution. This would be possible as the phases of signals for extrapolated (or interpolated) additional virtual receive elements can be calculated based on the evolution of phases of the actual signals into the receiver elements, using autoregressive methods.

In general formulation the autoregressive methods are able to predict, or extrapolate, future values of a sequence of {s} by analyzing its past values [36]:

(18) $$s\left[z\right]=-\sum_{w=1}^{W}a_{w}s\left[z-w\right]$$

where W is the AR model order, $a_{w}$ is the $w^{th}$ model coefficient, $z=H+1,\cdots ,N_{x}$, and $N_{x}$ is the total number of elements in the sequence after extrapolation is done.

The Burg algorithm can be used to estimate the values of coefficients, $a_{w}$, for either interpolation, when, for instance, some data are missing due to failure of array elements, or extrapolation, as in our case. The principle of the iterative-based BA operation is based on the minimization of the combined estimation error, $E_{i}$, through forward and backward predictions:

(19) $$E_{i}={f_{i}}^{†}f_{i}+{b_{i}}^{†}b_{i}$$

where *i* is the iteration number, ${\left(\cdot \right)}^{†}$ is the conjugate transpose, and $f_{i}$ and $b_{i}$ are forward and backward prediction error vectors, respectively.

The algorithm to produce the FIR filter coefficients, in other words, model coefficients, in (18), can be written as follows [26,27,28,29,37]:

Input:

- The input signal vector: $s={\left[s_{0},s_{1},s_{2},\cdots ,s_{H-1}\right]}^{T}$ where H is the number of input samples.
- AR model order: W.
- Desired total data length after extrapolation: $N_{x}\geq H$.

Output:

- AR coefficient vector: $a^{\left(W\right)}={\left[1,a_{1},a_{2},\cdots ,a_{W}\right]}^{T}$.

1. Initialization
  (20) $$f_{0}^{\prime }=s$$
  (21) $$b_{0}^{\prime }=s$$
2. Burg Iteration (for i=1:W)
  (a) Remove the first element of $f_{i}^{\prime }$ and the last element of $b_{i}^{\prime }$ in each iteration so that
  (22) $$f_{i}=f_{i}^{\prime }\left(1:H-i-1\right)\;\&\;b_{i}=b_{i}^{\prime }\left(0:H-i-2\right)$$
  (b) Compute the reflection coefficient ($k_{i}$):
  (23) $$k_{i}=-\frac{2{b_{i}}^{†}f_{i}}{{f_{i}}^{†}f_{i}+{b_{i}}^{†}b_{i}}$$
  (c) Update both forward and backward prediction vectors:
  (24) $$f_{i+1}^{\prime }=f_{i}+{k_{i}b}_{i}\;\&\;b_{i+1}^{\prime }=b_{i}+{{k_{i}}^{*}f}_{i}$$
  where ${\left(\cdot \right)}^{*}$ denotes the complex conjugate.
  (d) Calculate and update the model coefficient:
  (25) ai+1=ai0+kiYai*0, Y=0  …    0    1 ⋮          1     00                 ⋮1   0   …    0(i+1)×(i+1)

The choice of the model order, W, has a significant impact on the image quality. Using a low model order (e.g., W<H/4) may cause the Burg algorithm to inadequately represent the data; consequently, closely located targets may not be properly resolved, which limits the expected resolution enhancement. In contrast, using a high model order (e.g., W≅H−1) may produce spurious peaks between closely positioned targets and thus degrade image quality. Therefore, the model order must be carefully selected to preserve the image quality whilst achieving the resolution improvement. In this work, a model order of W=H/2 is used in all simulations and experimental validations.

## 3. Proposed Methods

As has already been mentioned, the combination of MIMO and DBS/SAR allows both the improvement in MIMO resolution in the lateral direction and suppression of MIMO sidelobes. To improve the resolution in the boresight direction, we will apply BA to both MIMO-DBS and MIMO-SAR and investigate its overall performance.

### 3.1. Burg-Aided MIMO-DBS

In this approach, at first, the range compression is done as in (11); then, the BA is applied to extrapolate data in the Doppler dimension. Then, a Doppler FFT is taken and phase shifts in MIMO arising from the motion of the radar platform in the case of TDM (15) are compensated as below:

(26) $$F_{B}\left(r,p,m\right)=\left(\sum_{n=1}^{\varepsilon_{N}N}D_{B}\left(r,n,m\right)e^{\frac{-j2\pi np}{\varepsilon_{N}N}}\right)e^{-j\Delta \beta_{m}}$$

where $D_{B}\left(r,n,m\right)$ is the extrapolated data along the Doppler dimension, $\varepsilon_{N}$ is the extrapolation factor used along the Doppler dimension, and $\Delta \beta_{m}$ is the phase shift of the $m^{th}$ virtual element in TDM.

Then the BA is used again to extrapolate the data in angular dimension. An FFT along the angular dimension is taken to obtain image domain 3-D data cube, $F_{BB}\left(r,p,q\right)$, as

(27) $$F_{BB}\left(r,p,q\right)=\sum_{m=1}^{\varepsilon_{M}M}F_{B}\left(r,p,m\right)e^{\frac{-j2\pi mq}{\varepsilon_{M}M}}$$

where $\varepsilon_{M}$ is the extrapolation factor along the angular dimension.

**Figure 5:**
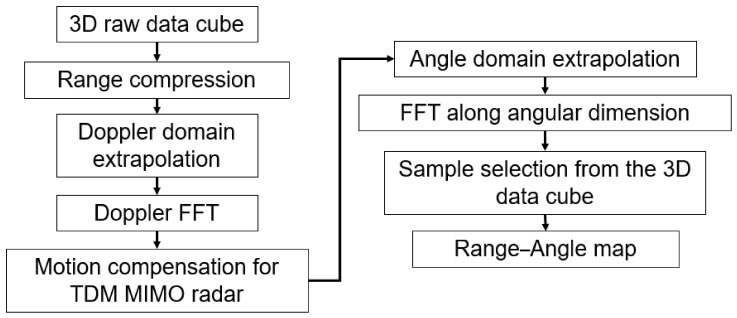
Flow chart of Burg-aided MIMO-DBS.

Finally, to form a range–angle map, samples are selected from the 3-D data cube at points where MIMO and DBS angles intersect, as described in Section 2.3. The flowchart of the BA MIMO-DBS data processing is shown in Figure 5. With two extrapolations by factors $\varepsilon_{M}$ and $\varepsilon_{N}$, an angular resolution as in (13) will change to

(28) $$\theta_{-3dB_{BB}}\approx \frac{0.9}{\sqrt{{\left(\frac{\varepsilon_{M}\left(M-1\right)d_{r}}{\lambda }cos\theta \right)}^{2}+{\left(\frac{2\varepsilon_{N}Tv_{p}sin\theta }{\lambda }\right)}^{2}}}$$

It is important to note that each extrapolation is done independently for each range bin. Since there is no interdependence between the range bins, this makes it possible to execute the extrapolation operations in parallel, so that Burg-aided processing can be significantly accelerated.

### 3.2. Burg-Aided MIMO-SAR

Here, the back-projection algorithm is applied, following the range compression of 3-D raw data, consisting of range bins, virtual elements and MIMO frames. However, instead of directly summing the contributions of each sample point as described in Section 2.4, each value of an internal expression in (16) for each (m,n) of Doppler and virtual array dimensions is calculated and stored. Therefore, at this point, the data becomes 4-dimensional, and the order of such an array is defined by the number of range bins, number of cross-range bins, number of virtual elements, and number of MIMO frames. The data can be represented as

(29) $${S^{\prime }}_{m,n}\left(k,l\right)=D\left(\left[R_{klmn}\right],m,n\right)e^{-i\frac{4\pi }{\lambda }R_{klmn}}$$

Here, each $\langle k,l\rangle$ pixel contains (M×N) complex values for all Doppler and virtual array sampling points. The number of pixels in the image is *M* × *N*:

(30) $$\langle k,l\rangle ={\left[\begin{array}{ccc} \begin{array}{c} m_{1}n_{1}\\ m_{2}n_{1} \end{array} & \ldots & \begin{array}{c} m_{1}N\\ m_{2}N \end{array}\\ \vdots & \ddots & \vdots \\ Mn_{1} & \ldots & MN \end{array}\right]}_{M\times N}$$

The Burg algorithm is subsequently used to extrapolate the data along both Doppler and virtual array dimensions at each pixel of the image. Hence, after extrapolation, (30) increases to

(31) $${\langle k,l\rangle }_{Burg}={\left[\begin{array}{ccc} \begin{array}{c} m_{1}n_{1}\\ m_{2}n_{1} \end{array} & \ldots & \begin{array}{c} m_{1}N\varepsilon_{N}\\ m_{2}N\varepsilon_{N} \end{array}\\ \vdots & \ddots & \vdots \\ M\varepsilon_{M}n_{1} & \ldots & M\varepsilon_{M}N\varepsilon_{N} \end{array}\right]}_{M\varepsilon_{M}\times N\varepsilon_{N}}$$

Then, a 2-D range–cross-range image is generated by coherently summing the values at each pixel as

(32) $$S{\left(k,l\right)}_{Burg}=\sum_{m=1}^{M\varepsilon_{M}}\sum_{n=1}^{N\varepsilon_{N}}{S^{\prime }}_{m,n}{\left(k,l\right)}_{Burg}\;$$

The achievable angular resolution in the BA MIMO-SAR is the same as the theoretical resolution given by (28).

**Figure 6:**
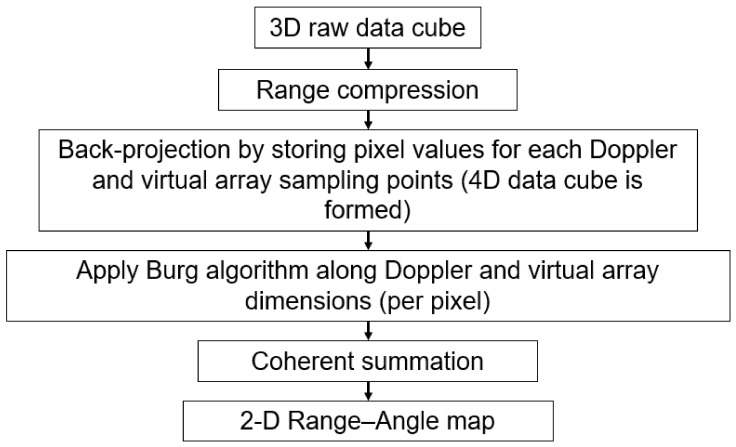
Flow chart of Burg-aided MIMO-SAR.

The flowchart of this method is shown in Figure 6. Table 2 contrasts BA MIMO-DBS and BA MIMO-SAR. The performances of both approaches under the RCM, phase variation and non-linear platform motion are the same as in the cases of MIMO-DBS and MIMO-SAR. However, applying BA to the MIMO-SAR requires significantly higher random-access memory (RAM) since 4-dimensional data is to be stored. Therefore, as the imaged area expands or images are formed with higher resolutions, the increase in the number of pixels leads to a substantial increase in RAM. Consequently, the imaged area often needs to be divided into smaller sub-images that are processed separately and then combined to form a larger image. Conversely, the BA MIMO-DBS technique does not require as much memory due to the inherent characteristics of the DBS algorithm.

Therefore, the time to form the resolution-enhanced image with BA MIMO-SAR takes much longer than that of BA MIMO-DBS due to the application of BA to each pixel of the imaged area, as will be shown in the next sub-section.

### 3.3. Computational Complexity

In this section, the computational complexity (CC) of each method, starting from the DBS technique and extending to the proposed methods, will be presented and analyzed to demonstrate notable differences in CC across the various methods.

For the DBS technique, the primary computational load arises from the FFT operations. Assuming $N_{f}$ range samples and N Doppler pulses, the CC is as follows:

- $O\left(N_{f}N\log\left(N_{f}\right)\right)$ for range compression;
- $O\left(N_{f}N\log\left(N\right)\right)$ for Doppler processing.

Consequently, the required CC for the DBS technique is $O\left(N_{f}N\log\left(N_{f}N\right)\right)$.

In the case of the MIMO-DBS technique, an additional azimuth processing step is included, along with the range compression and Doppler processing steps, which are the same as in the DBS technique. Therefore, the computational complexity of the MIMO-DBS technique can be estimated as follows:

- $O\left(N_{f}NM\log\left(N_{f}\right)\right)$ for range compression;
- $O\left(N_{f}NM\log\left(N\right)\right)\;$ for Doppler processing;
- $O\left(N_{f}NM\log\left(M\right)\right)\;$ for azimuth processing.

Hence, in total, the CC for the MIMO-DBS technique is $O\left(N_{f}NM\log\left(N_{f}NM\right)\right)$. For simplification, assuming $N_{f}=N=M=$ Q, the total CC is estimated as $O\left(Q^{3}\log\left(Q\right)\right)$.

For the back-projection approach in MIMO-SAR, the number of operations can be estimated as $O\left(N_{f}NMP\right)$, where $P=N_{r}N_{cr}$, and where $N_{r}$ is the number of range pixels and $N_{cr}$ is the number of cross-range pixels. With similar assumptions as before ($N_{f}=N=M=$ Q), the CC of BP can be simply expressed as $O\left(Q^{3}P\right)$ and, considering that the value of P is very high compared to other parameters, the CC of MIMO-SAR becomes significantly higher than that of MIMO-DBS. For clarity, this relationship can be expressed as $O\left(Q^{3}\log Q\right)\ll O\left(Q^{4}\right)<O\left(Q^{3}P\right)$.

Regarding the computational complexity of the Burg-aided MIMO-DBS technique, assuming $p_{D}$ and $p_{A}$ are the orders of the filter in (18) for extrapolating the data along the Doppler and angular dimensions, the number of operations are:

- $O\left(N_{f}NM\log\left(N_{f}\right)\right)$ for the range compression;
- $O\left(N_{f}Mp_{D}(\varepsilon_{N}N)\log\left(\varepsilon_{N}N\right)\right)\;$ for extrapolation along the Doppler and the Doppler processing;
- $O\left(N_{f}\left(\varepsilon_{N}N\right)p_{A}(\varepsilon_{M}M)\log\left(\varepsilon_{M}M\right)\right)$ for extrapolation along azimuth and the azimuth FFT.

Hence, the total computation time of BA MIMO-DBS is $O(N_{f}NM\log N_{f})+O(N_{f}NM(p_{D}\varepsilon_{N}\log\left(\varepsilon_{N}N\right)+\varepsilon_{N}\varepsilon_{M}p_{A}\log\left(\varepsilon_{M}M\right)))$.

For ease of comparison, by assuming that $p_{D}=p_{A}=p_{B}$ and $\varepsilon_{N}=\varepsilon_{M}=\varepsilon$, the total CC is estimated as $O\left(Q^{3}\log Q\right)+O\left(Q^{3}p_{B}\varepsilon \log(\varepsilon Q\right)(1+\varepsilon ))$.

Finally, for the BA MIMO-SAR, the number of operations are:

- $O\left(N_{f}NMP\right)$ for back-projection;
- $O\left(PMp_{D}(\varepsilon_{N}N)\right)$ for extrapolation along the Doppler dimension;
- $O\left(P\left(\varepsilon_{N}N\right)p_{A}(\varepsilon_{M}M)\right)$ for extrapolation along azimuth dimension.

As the result, the total computational complexity of Burg-aided MIMO-SAR is $O\left(N_{f}NMP\right)+O(PNM\left(p_{D}\varepsilon_{N}+p_{A}\varepsilon_{N}\varepsilon_{M}\right))$ and its simplified version is $O(Q^{3}P)+O\left(PQ^{2}p_{B}\varepsilon (1+\varepsilon )\right)$, which is significantly higher than that of BA MIMO-DBS.

## 4. Simulation Results

In this section, we examine and compare the performance of MIMO, DBS, MIMO-DBS, MIMO-SAR, BA MIMO-DBS and BA MIMO-SAR techniques using simulation.

**Figure 7:**
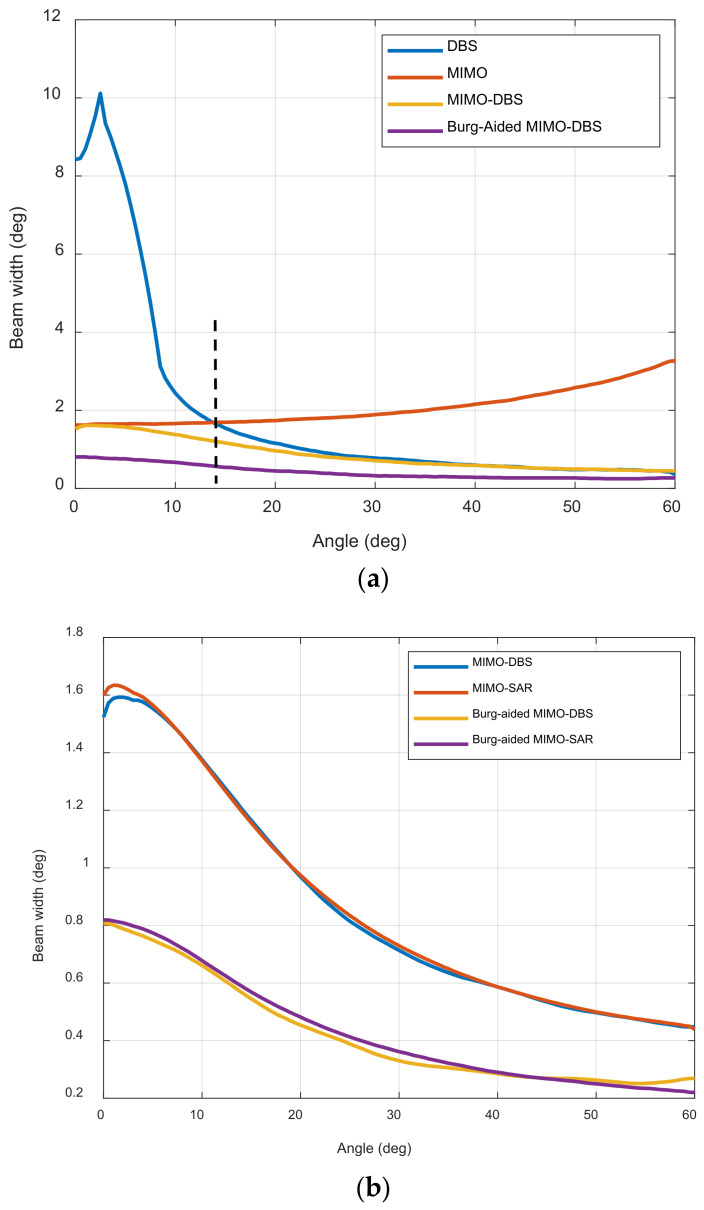
Achieved angular resolutions of (**a**) DBS, MIMO, MIMO-DBS and BA MIMO-DBS [26], (**b**) MIMO-DBS, MIMO-SAR and their Burg-aided ones.

First, the achievable −3 dB angular resolutions as a function of azimuth look-angle, obtained by each of the considered methods, are shown in Figure 7 using the radar parameters listed in Table 3. In this illustrative example, the velocity of the radar platform is 2 m/s, and the total coherent integration time is chosen to be 128 ms. A bandwidth of 500 MHz is used to avoid RCM, as in (10). Figure 7a shows that with the increase of the angle to the target, the angular resolution in the case of MIMO beamforming coarsens, but improves in the case of DBS. For the given speed of the platform, from (14), the transition point $\theta^{\prime }$ of the MIMO-DBS technique is calculated as 13.3^0^, indicated by a dashed vertical line, after which the use of the combined MIMO-DBS technique improves the angular resolution, compared to MIMO. In the case of BA MIMO-DBS with a Burg extrapolation factor equal to 2 for both dimensions, there is a further two-times refinement of both on- and off-boresight resolution.

Figure 7b shows that very similar resolutions are obtained for MIMO-SAR and MIMO-DBS and their BA versions, as was previously explained in Section 3.1 and Section 3.2.

**Figure 8:**
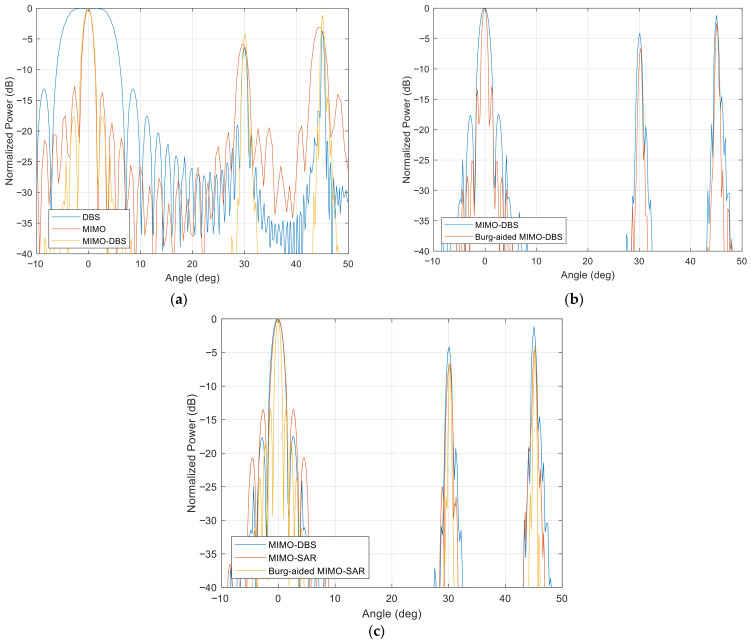
Azimuth profiles of different RCS targets for (**a**) DBS, MIMO, MIMO-DBS, (**b**) MIMO-DBS and BA MIMO-DBS, (**c**) MIMO-DBS, MIMO-SAR and BA MIMO-SAR.

Figure 8 shows the improvement in the resolutions and decreased level of the MIMO sidelobes for MIMO and DBS in Figure 8a, and MIMO-DBS and MIMO-SAR, as well as their BA extrapolation in Figure 8b,c. Here, three targets are located at 0^0^, 30^0^, and 45^0^ azimuth angles at the same range and zero elevation angle in the far-field region of the radar with the parameters as described before. The off-boresight targets have 6 dB and 3 dB less RCS than the boresight targets, respectively. The angular response of MIMO-DBS and MIMO-SAR is equal to MIMO’s resolution at 0^0^, while a twice-higher resolution is attained by implementing the BA in both the Doppler and angle dimensions. Furthermore, the angular resolution improvement in off-the-boresight targets is seen in both BA MIMO-DBS and BA MIMO-SAR, compared to MIMO-DBS/MIMO-SAR.

**Figure 9:**
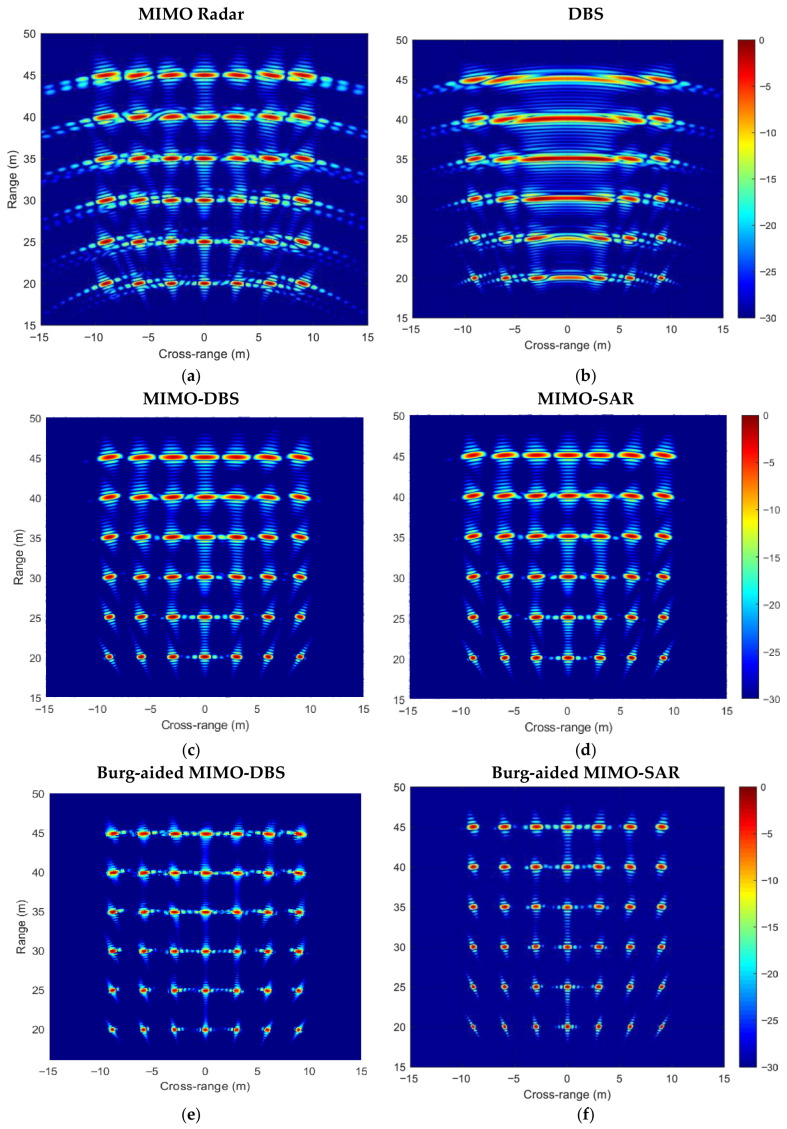
Range–cross-range maps of (**a**) MIMO radar, (**b**) DBS, (**c**) MIMO-DBS, (**d**) MIMO-SAR, (**e**) Burg-aided MIMO-DBS, (**f**) Burg-aided MIMO-SAR. Bandwidth is 500 MHz.

The performance of all the techniques is further illustrated in 2-D range–cross-range maps in Figure 9. Here, 42 point-like targets at zero elevation with respect to radar are placed with a separation of 3 m from −9 m to 9 m in cross-range and with a separation of 5 m in range in the region from 20 m to 45 m. The radar parameters and platform velocity are the same as in the previous scenarios. For easy visual comparison of the resolution, the power decrease related to the path loss is compensated. A significant reduction in sidelobe levels and improvement in the resolution compared to MIMO and DBS in Figure 9a,b are seen in Figure 9c after the compensation for the phase error in TDM MIMO caused by the motion of the platform. The phase error, Δβ, in (15) is compensated by multiplying the range compressed data by $e^{-j\Delta \beta }$.

The MIMO-SAR image is shown in Figure 9d by applying BP to the range-compressed data with the use of the positional information of the radar to form the phase-error-free image.

Twice-improved angular resolution in both the forward and lateral directions can be seen in both BA MIMO-DBS and BA MIMO-SAR maps in Figure 9e and Figure 9f, respectively.

**Figure 10:**
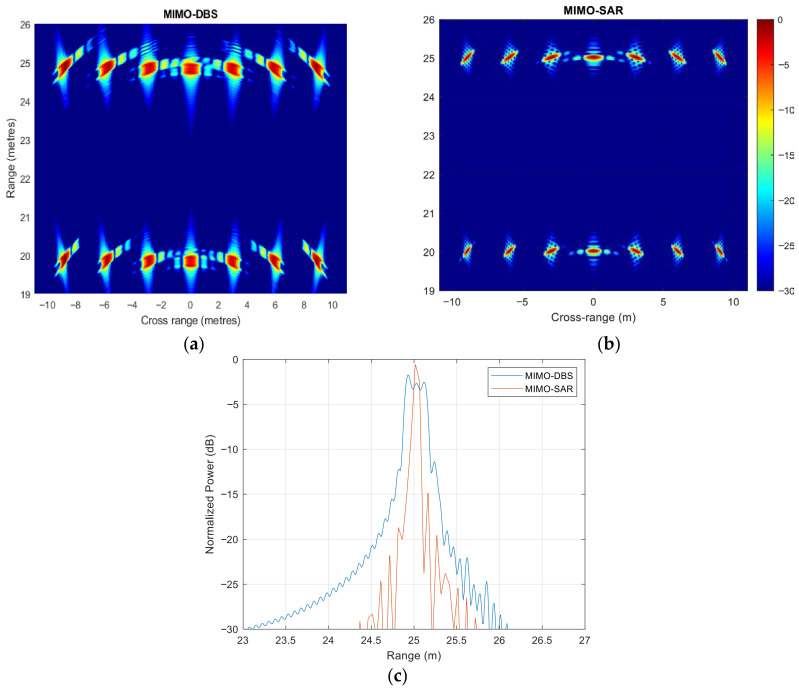
(**a**) MIMO-DBS and (**b**) MIMO-SAR, (**c**) zero cross-range cut of both MIMO-DBS and MIMO-SAR techniques for the bandwidth of 2 GHz.

In all previous simulations, the synthetically created aperture length is less than the range resolution of 30 cm, so that there is no RCM. However, enhancement of resolution in all domains, including range, is the main goal of the mm-wave radar designed for mobile platforms, specifically for vehicles. Therefore, the effect of RCM on MIMO-DBS was assessed by increasing the bandwidth to 2 GHz, while maintaining the other radar parameters constant. For the MIMO radar, looking in a forward direction, when data is collected at a constant velocity of 2 m/s, the synthetic aperture traverses four range cells for boresight targets, according to (10). The simulation results for MIMO-DBS and MIMO-SAR are then shown in Figure 10a,b, respectively, for two lines of targets at the ranges of 20 m and 25 m. Due to the higher bandwidth, the range resolution is improved by a factor of four and the MIMO-SAR does not suffer a degradation in performance. However, this increased bandwidth in MIMO-DBS leads to an energy spread across traversed range cells, resulting in an unfocused radar image, as demonstrated in Figure 10c. To overcome RCM, as stated in Section 2, either a specialized RCMC algorithm or weightings can be used.

**Figure 11:**
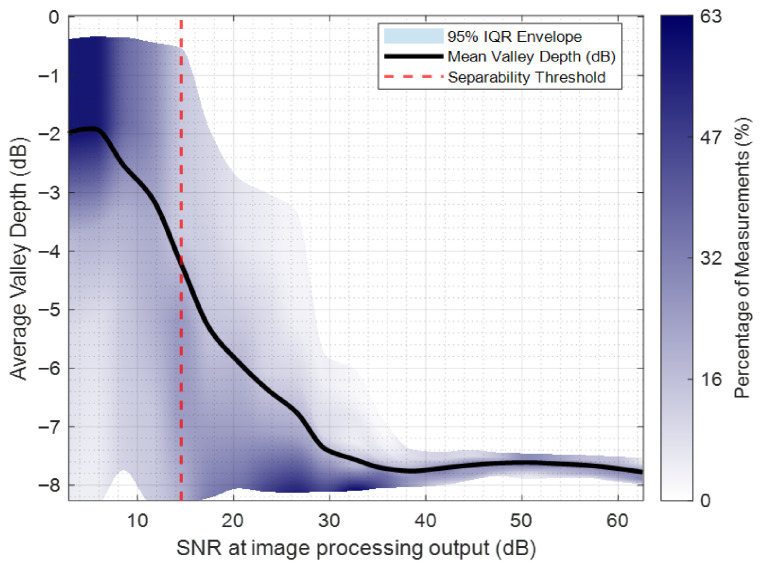
Average valley depth versus SNR at image processing output for two closely spaced targets. The red dashed line indicates the empirical separability threshold.

Another important parameter is the signal-to-noise ratio (SNR). Therefore, in the following analysis, the performance of the Burg algorithm is evaluated using Monte Carlo simulations with 250 trials per SNR level. The analysis examines the valley depth between two closely spaced targets that are unresolved before extrapolation, as well as how their separability improves with increasing SNR. Figure 11 shows the average valley depth across trials as a function of the SNR of the formed images after adding the processing gains. The valley region is defined using the 3 dB points of each target in the noise-free case, and this fixed interval is used to compute the average power (valley depth) for each trial.

Images formed are normalized with respect to the noise-free realization. The shaded area shows the 95% interquartile range (IQR), indicating the variability over trials. The density of estimations is shown by a dark-to-light blue gradient. As the SNR increases, the uncertainty in the average valley depth decreases, and the probability of observing lower valley depths increases. Beyond 32 dB SNR, values cluster around −8 dB, closely matching the noise-free case. The solid black curve represents the mean valley depth, which declines from −2 dB to approximately −8 dB as SNR increases from 10 to 35 dB.

Regarding the vertical red dashed line in Figure 11, it indicates the empirical separability threshold at roughly 15 dB SNR. Beyond this SNR value, the two targets become resolvable. Independently derived from an estimator-based analysis, this threshold corresponds to the SNR where the 95% confidence intervals of the estimated positions no longer overlap, therefore demonstrating consistent separation based on target location estimates.

The Monte Carlo simulations illustrate that the Burg algorithm offers significantly better resolution in expectation, compared to conventional FFT based methods. The Burg algorithm slightly improves peak-to-sidelobe ratio (PSLR) and integrated sidelobe ratio (ISLR) in expectation when SNR is low to moderate. However, at high SNR (e.g., 35 dB), the two methods—Burg algorithm-based and FFT-based methods—show similar sidelobe measures.

## 5. Experimental Results

In this section, the performance of MIMO-DBS and MIMO-SAR is evaluated through both laboratory-based and real-world experiments.

### 5.1. Lab-Based Experiments

The experiments have been conducted at the School of Engineering, University of Birmingham. The experimental data has been collected using INRAS Radarbook, with the MIMO radar operating at 77 GHz [38]. It has 4 Tx and 8 Rx elements aligned in azimuth, which provides 29 virtual elements with λ/2 spacing due to the overlapping of three elements. During the experiments, 0.5 GHz and 2 GHz bandwidths were used, giving a range resolution of 30 cm and 7.5 cm, respectively. The radar parameters are detailed in Table 4.

**Figure 12:**
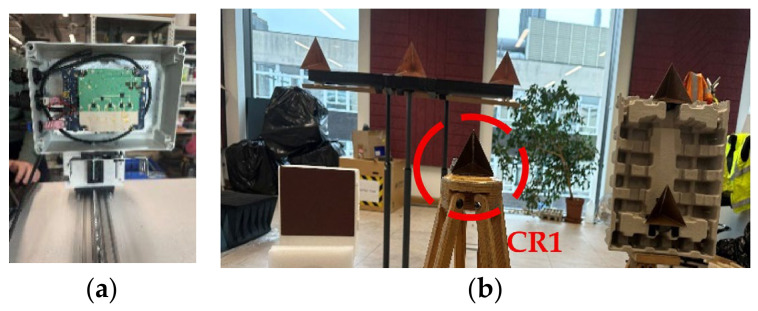
(**a**) 77 GHz INRAS MIMO radar on the linear positioner used, (**b**) calibrated targets.

**Figure 13:**
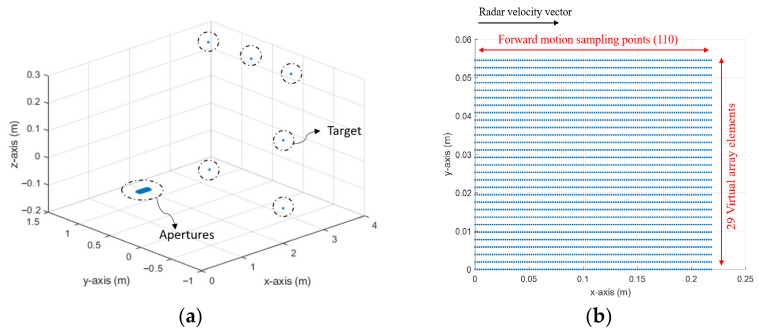
(**a**) The experimental scenario, (**b**) collected data points during the experiments.

The experimental setup is shown in Figure 12. Six triangular corner reflectors and a rectangular plate were used as targets. One corner reflector, CR1, with 7 cm edges was positioned 1.7 m away along the boresight, as shown in Figure 13. The rectangular target was placed 2.5 m away along the ground range and 0.8 m along the cross-range. At the same ground range of 2.5 m, two corner reflectors with 10 cm edges were placed at an azimuth angle of 15° with separation of 0.36 m in elevation. The other three corner reflectors, each with 15 cm edges, were positioned 3.7 m along the ground range and 0.25 m in elevation, spaced 0.7 m and 0.65 m apart in azimuth.

The radar platform was moved forward on a linear motion positioner (Figure 12). The velocity of the platform was 1.6 m/s, allowing the radar to collect data at 2 mm intervals. With 110 sweeps, the total aperture length is 22 cm. The mutual positions of targets and synthetic radar apertures of VA elements are shown in Figure 13.

**Figure 14:**
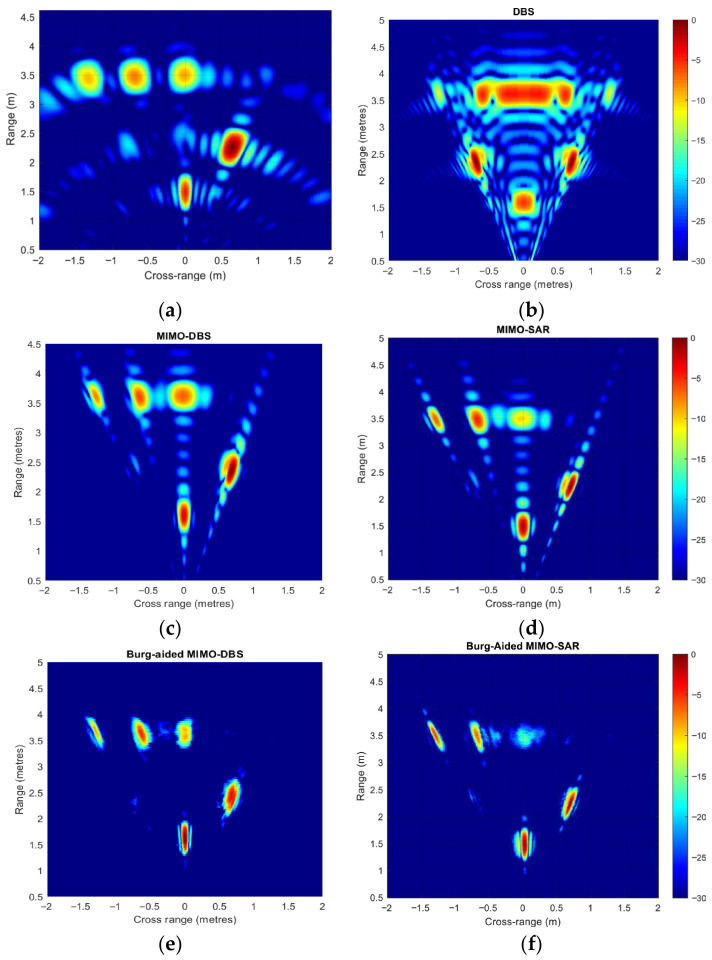
The results of lab-based experiments using 500 MHz bandwidth (**a**) MIMO radar, (**b**) DBS, (**c**) MIMO-DBS, (**d**) MIMO-SAR, (**e**) Burg-aided MIMO-DBS, (**f**) Burg-aided MIMO-SAR.

Figure 14 shows radar maps corresponding to the discussed techniques with the bandwidth of the signal at 500 MHz. While the high sidelobes can be seen in the MIMO map in Figure 14a, the DBS map in Figure 14b shows a notable angular improvement in the off-boresight directions, but there is an ambiguity in the angular positions of targets relative to the boresight, which is then resolved in MIMO-DBS maps in Figure 14c, demonstrating improved angular resolution off-boresight with significantly suppressed sidelobe levels. MIMO-SAR produces a response very similar to MIMO-DBS, with differences of only a few dB in the peak power of the targets, as illustrated in Figure 14d. The BA MIMO-DBS and BA MIMO-SAR maps shown in Figure 14e and Figure 14f, respectively, were obtained by extrapolating the radar data collected in both the Doppler and angular directions with the factor two by use of the Burg algorithm, so that the number of sweeps is increased from 110 to 220, and the number of virtual array elements is extrapolated from 29 to 58. Both techniques show twice-improvement in cross-range resolutions in both forward and lateral directions. BA MIMO-SAR produces more focused image, but the intensity of the boresight target at a distance of 3.7 m is slightly less.

**Figure 15:**
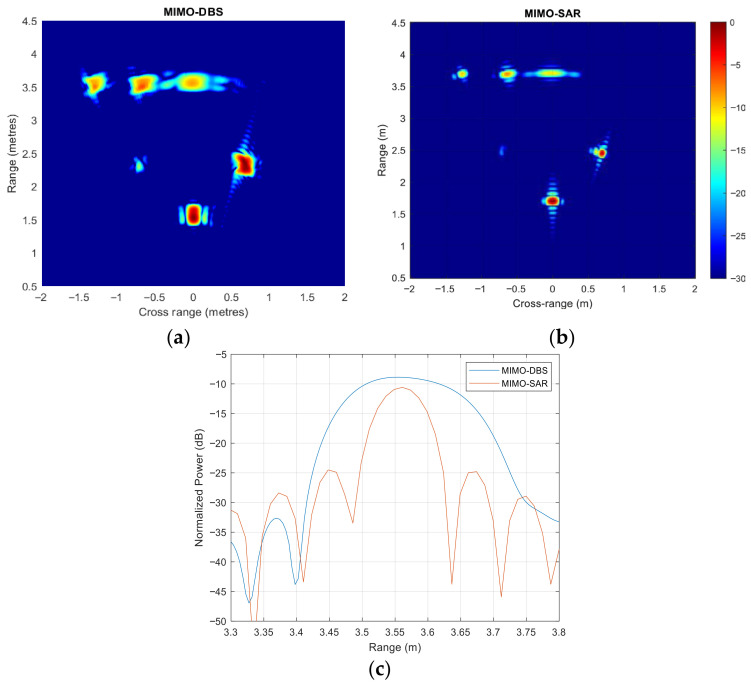
The results of lab-based experiments using 2 GHz bandwidth (**a**) MIMO-DBS, (**b**) MIMO-SAR, (**c**) zero cross-range cut of both MIMO-DBS and MIMO-SAR techniques.

The experiment was repeated with a bandwidth of 2 GHz, while keeping all other parameters from Table 4 unchanged, to assess the impact of RCM. As expected, this increase in bandwidth resulted in a less focused MIMO-DBS map, shown in Figure 15a, compared to the MIMO-SAR result in Figure 15b, with the comparison of results in Figure 15c.

### 5.2. Real-World Experiments

The real-world experimental data has been collected using a 77 GHz INRAS RadarLog MIMO radar with 2 GHz bandwidth [39] at the campus of the University of Birmingham. Four Tx and 16 Rx allow formation of 61 virtual linear elements aligned in azimuth with three virtual elements overlapping. The vehicle’s kinematics across 6 degrees of freedom (positions and orientations) was captured using a fiber optic gyroscope Inertial Measurement Unit (IMU) [40].

**Figure 16:**
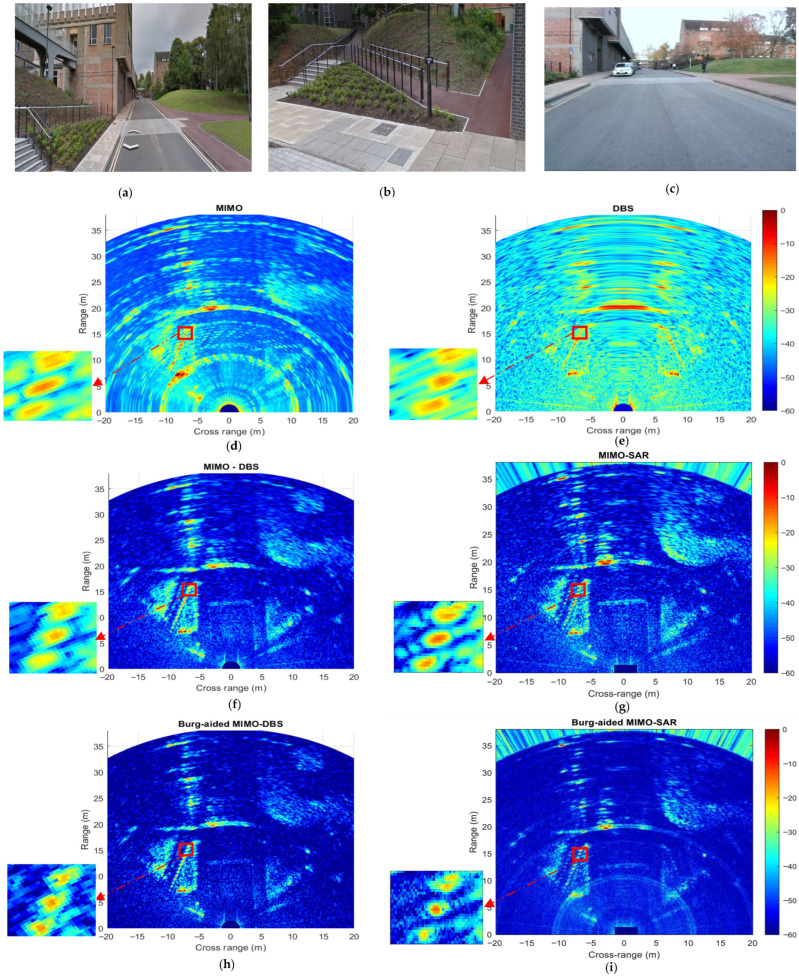
The results of the first real-world experiment using 2 GHz bandwidth: (**a**) Google Earth image of the experimental scene, (**b**) Google Earth image of the railing area on the left-hand side of the experimental scene, (**c**) picture of experimental scene, (**d**) MIMO radar, (**e**) DBS, (**f**) MIMO-DBS, (**g**) MIMO-SAR, (**h**) BA MIMO-DBS, and (**i**) BA MIMO-SAR.

The experimental scene is shown using Google Earth images in Figure 16a,b, and a photograph taken during the data recording in Figure 16c. The radar parameters used are shown in Table 5. Compensation of phase errors for each VA element of TDM MIMO is performed as described in Section 4, and the resultant MIMO is shown in Figure 16d. While the regularly spaced railings on the left-hand side of the scene and the car in front are visible, the fine details are not discernible due to the low angular resolution and high sidelobes.

In the DBS technique, the total aperture length, $CPI\times v_{p}$, is equal to 19.2 cm, which is almost four times larger than the range resolution. Therefore, to reduce the RCM effects in this experiment, a Blackman–Harris weighting, which has superior sidelobe suppression ability compared to other windows, is implemented in the range dimension before the range compression, inevitably at the cost of coarsening the resolution. While there is an expected improvement in cross-range resolution off-boresight, the left–right ambiguity of the actual positions of the targets is evident, which also leads to an elevated background level, as seen in Figure 16e.

In MIMO-DBS processing, with the result shown in Figure 16f, a Blackman–Harris weighting is again applied before range compression; then, a Hann weighting is utilized prior to Doppler processing and MIMO beamforming to minimize spectral leakage produced by the FFT. The MIMO-DBS map shows very detailed scene representation with clearly discernible segments of such a “clutter map” consisting of car, railings, road, grass, pavement and a speed bump, owing to sidelobe suppression.

In MIMO-SAR, the collected radar data is directly processed using the recorded IMU data without any additional phase error corrections as in MIMO-DBS. Figure 16g shows higher focusing of returns from the scene than in the case of the MIMO-DBS, where interpolation is required to select the intersecting samples along both the MIMO and DBS dimensions, as mentioned in Section 2.3. It is worth noting, however, that for the path planning, the radar image segmentation based on the contrast of returns from different regions must be performed in real time [41]; therefore, the much faster MIMO-DBS would be a preferred technique for automotive applications.

The results of the proposed approaches—BA MIMO-DBS and BA MIMO-SAR, shown in Figure 16h and Figure 16i, respectively—demonstrate the cross-range resolution improvement by a factor of two due to BA-aided extrapolation. However, significant differences in the background level are observed here: the BA MIMO-DBS approach yields a further notable reduction in background level, thereby enhancing the visibility of road-related infrastructure details such as railings, pathways behind the railings, and grass areas, rendering them more discernible. In contrast, the BP used to generate a BA MIMO-SAR image causes an increase in the background level and the discernibility of details such as speed bumps and grass reduces, as their reflections merge with the background level.

**Figure 17:**
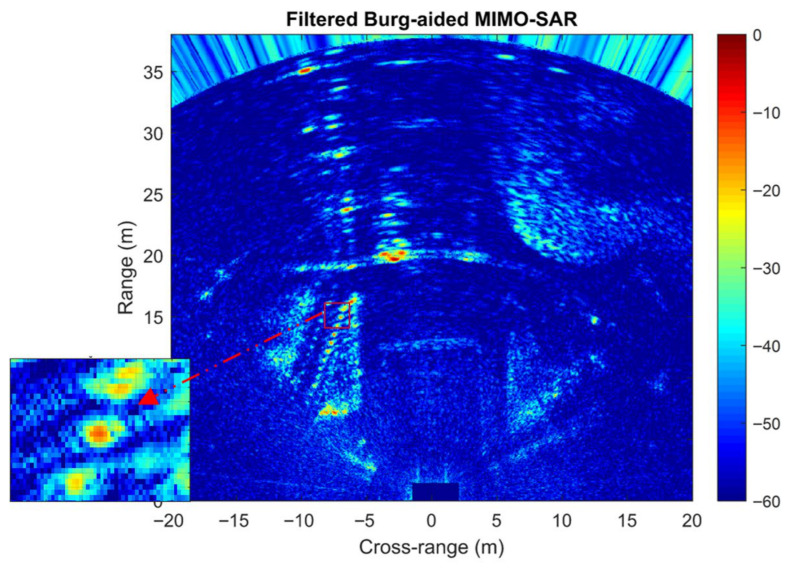
Filtered response of Figure 16g,i.

To extract more detailed information in Burg-aided MIMO-SAR, image filtering was implemented. This approach makes automatic comparison of the intensity values of each pixel in both the MIMO-SAR and Burg-aided MIMO-SAR results; then, the minimum intensity value among corresponding pixels is selected to construct the new image. Although this filtering method may attenuate the responses of low-reflectivity targets present in the scene, the result in Figure 17 shows both improved cross-range resolution and improved background levels, facilitating the extraction of finer details from the radar scene.

## 6. Conclusions

This study presents a systematic approach to analyze and compare the synthetic approaches leading to high-fidelity automotive radar imagery of road scenes ahead of vehicles, due to both high achievable resolution and sensitivity. Specifically it has been shown that the extrapolation of MIMO-DBS and MIMO SAR data across both Doppler and angular dimensions using the Burg algorithm can significantly enhance the angular resolution in both the forward and lateral directions of conventional MIMO automotive radar. The analysis of individual and combined approaches has been made to contrast and compare the benefits and limitations of each, specifically devising the estimates of the computational complexity and additional processing steps to address the discussed limitations. The results and achievable performance were demonstrated through both simulations and experiments in the lab and real-world conditions using 77 GHz MIMO radar.

## Figures and Tables

**Table 1:** Comparison of MIMO-DBS and back-projection.

	MIMO-DBS	BP
Range cell migration	Yes	No
Effect of phase variation	Yes	No
Effect of non-linear motion	Yes	No
Computational time	Small	High

**Table 2:** Burg-aided MIMO-DBS vs. Burg-aided MIMO-SAR.

	BA MIMO-DBS	BA MIMO-SAR
Range cell migration	Yes	No
Effect of phase variation	Yes	No
Effect of non-linear motion	Yes	No
RAM requirement	Small	High
Computational time	Small	High

**Table 3:** Radar Parameters.

Parameters	Value
Modulation	FMCW
Centre frequency	77 GHz
Bandwidth	0.5 GHz
Sample rate	10 MSa/s
Samples per chirp	2048
Chirp interval	230 μs
MIMO frame interval	1 ms
Frame interval	128 ms
Beamwidth at boresight	$1.7{}^{\circ}\;(61\;\mathrm{VA}\;\mathrm{with}\;d_{r}=\lambda /2$)

**Table 4:** Radar Parameters used in lab-based experiments.

Parameters	Value
Modulation	FMCW
Centre frequency	77 GHz
Bandwidth	0.5 GHz & 2 GHz
Tx/Rx number	4/8
Virtual array number	29
Chirp interval	256 μs
MIMO frame interval	1.25 ms
Total CPI	137.5 ms
Velocity of the radar	1.6 m/s
Step of data collection	2 mm

**Table 5:** Radar Parameters.

Parameters	Experiment
Modulation	FMCW
Centre frequency, GHz	77
Bandwidth, GHz	2
Tx/Rx number	4/16
Virtual array number	61
Beamwidth, degree	1.7
Chirp interval, μs	115
MIMO frame interval, ms	0.5
Frame interval, ms	64
Velocity of the radar, m/s	3

## Data Availability

Dataset available on request from the authors.
